# A New Medical Dictionary

**Published:** 1890-08

**Authors:** 


### Bibliographical.

A NEW MEDICAL DICTIONARY.—A new dictionary including all the words and phrases used in medicine, with their proper pronunciation and definitions, based on recent medical literature, by George M. Gould, B.A., M,D., Ophthalmic Surgeon to the Philadelphia Hospital, etc., with tables of the Bacilli, Micrococci, Leucomaïnes, Ptomaïnes, etc., of the Arteries, Muscles, Nerves, Ganglia and Plexuses; Mineral Springs of U. S., Vital Statistics, etc. Small octavo, 520 pages. Half dark leather, $3.25; Half Morocco, thumb index, $4.25. Philadelphia: P. Blakiston, Son & Co.

The object of this new Dictionary of Medicine is explained in the following extract from the preface:

1. To include those *New Words and Phrases* created during the past ten years—a period rich in coinages—which appeared destined to continuous usage. There are certainly thousands of these, and in their compilation I have especially endeavored to cover the latest results in the study of Bacteriology, Ptomaines, Leucomaines, Electrotherapeutics, Embryology, Physiology, Pathology, etc., and in the various special branches of medicine such as Ophthalmology, Otology, Laryngology, Gynæcology, etc.

2. To frame all *definitions* by the direct aid of *New Standard, and Authoritative Text-books,* instead of making a patchwork of mechanical copying from older vocabularies.

3. While neglecting nothing of positive value, to *omit obsolete words* and those not pertinent to medicine except in a remote or factitious sense.

4. To make a volume that will answer the needs of the medical student and busy practitioner, not only by its *compactness of arrangement* and *conciseness of definitions*, but also by its *convenience of size* and *price*.

A few dental mistakes may be noted: "Eye teeth" are defined *Incisor Teeth* instead of Canine Teeth, and "Premolars" are defined as the *Molar Teeth* instead of the Bicuspid Teeth, to which latter teeth the term *Premolars* is commonly applied. The condensed form of this dictionary which, notwithstanding, is very voluminous in words and definitions, will render it a very convenient, as well as useful, text-book.

